# Association between neutrophil-to-high-density lipoprotein cholesterol ratio and non-alcoholic fatty liver disease or metabolic dysfunction-associated steatotic liver disease: evidence from NHANES 2017–2020

**DOI:** 10.3389/fmed.2024.1491858

**Published:** 2025-01-15

**Authors:** Na Zhu, Yanyan Li, Yingying Lin, XinYu Cui, Xin Li

**Affiliations:** ^1^Center of Integrative Medicine, Beijing Ditan Hospital, Capital Medical University, Beijing, China; ^2^Center of Integrative Medicine, Peking University Ditan Teaching Hospital, Beijing, China

**Keywords:** non-alcoholic fatty liver disease, metabolic dysfunction-associated steatotic liver disease, neutrophil-to-high-density lipoprotein cholesterol ratio, inflammation, lipid metabolism disorders, liver fibrosis

## Abstract

**Background:**

Non-alcoholic fatty liver disease (NAFLD) or metabolic dysfunction-associated steatotic liver disease (MASLD) is closely associated with chronic inflammation and lipid metabolism disorders. The neutrophil-to-high-density lipoprotein cholesterol ratio (NHR) is an integrative marker reflecting inflammatory responses and lipid metabolism disorders and is associated with various diseases. This cross-sectional study aimed to determine the association between NHR and NAFLD, MASLD, and liver fibrosis.

**Methods:**

Data for this study were obtained from the 2017–2020 National Health and Nutrition Examination Survey (NHANES), we employed weighted multiple regression and restricted cubic spline (RCS) analysis to assess the relationship between NHR and NAFLD, MASLD, and liver fibrosis. Additionally, we performed stratified analyses based on gender, age, body mass index, diabetes, hypertension, smoking status, and history of cardiovascular disease to evaluate the consistency of these associations across different subgroups.

**Results:**

A total of 6,526 participants were included in the study. 2,839 (weighted 44.1%) participants were diagnosed with NAFLD and 2,813 (weighted 43.7%) participants were diagnosed with MASLD. After adjusting for confounders, NHR was positively associated with the risk of NAFLD/MASLD, and the correlation was particularly significant in the subgroups of females, those without hypertension, and those without diabetes (*p* < 0.05). By the NHR quartile, the risk of NAFLD/MASLD increased progressively with higher NHR levels (P for trend <0.001). In addition, RCS analysis showed a nonlinear association between NHR and NAFLD/MASLD and liver fibrosis (P-non-linear <0.05).

**Conclusion:**

NHR may serve as a potential marker for NAFLD/MASLD and liver fibrosis, and lowering NHR levels could help reduce the incidence of these conditions.

## Introduction

As modern lifestyles change, the incidence of metabolic diseases such as obesity and diabetes continue to rise, and the closely related problem of liver disease is becoming more and more prominent. Among them, non-alcoholic fatty liver disease (NAFLD) has become an important burden on global public health, affecting about a third of the world’s adult population (1, 2). As NAFLD is closely associated with multiple metabolic dysfunctions, to characterize this disease more accurately, in June 2023, an international consensus of experts decided to rename NAFLD as MASLD, emphasizing the central role of cardiometabolic risk factors in the development of the disease (3). The pathological features of NAFLD/MASLD include hepatocellular steatosis, inflammatory cell infiltration, and different degrees of hepatic fibrosis (4). For patients who have developed advanced hepatic fibrosis, the leading causes of death are liver-related complications and extrahepatic malignancies (5). For patients with NAFLD/MASLD who have not developed liver fibrosis, cardiovascular disease is the leading cause of death (6). Therefore, early detection of NAFLD/MASLD and liver fibrosis is crucial for timely intervention and effective management.

Inflammatory response and abnormal lipid metabolism are critical in causing and developing NAFLD/MASLD (7). As an essential component of the innate immune system, neutrophils play a central role in fighting infections. They are closely associated with various chronic inflammatory diseases, especially metabolic diseases such as obesity, type 2 diabetes, and NAFLD/MASLD (8). Several studies have shown that the degree of neutrophil infiltration in liver tissue of NAFLD/MASLD patients is positively correlated with the severity of the lesion. Neutrophil infiltration can directly lead to hepatocellular injury and accelerate the progression of NAFLD/MASLD to hepatic fibrosis and cirrhosis by promoting fibrosis (7–9). NAFLD/MASLD begins with excessive accumulation of triglycerides in the hepatocytes and is accompanied by a reduction in plasma cholesterol levels associated with antiatherosclerosis high-density lipoproteins (10). HDL-C is critical in developing NAFLD/MASLD through lipid metabolism modulation and reverse cholesterol promotion. HDL-C exerts anti-inflammatory and antioxidant effects by regulating lipid metabolism, promoting reverse cholesterol transport, and reducing the production of inflammatory mediators (11). Therefore, reducing HDL-C levels in NAFLD/MASLD may weaken its anti-inflammatory and antioxidant functions and thus exacerbate the occurrence and progression of NAFLD/MASLD (12). In recent years, NHR has been proposed as a comprehensive indicator of inflammation and lipid metabolic status. It has been shown that NHR is significantly associated with cardiovascular disease, metabolic syndrome, hepatocellular carcinoma, and Parkinson’s disease (13–16), but its potential relationship with NAFLD/MASLD has not been fully investigated.

This study used data from the 2017–2020 National Health and Nutrition Examination Survey (NHANES) in a cross-sectional analysis to investigate the association between NHR and both NAFLD/MASLD and liver fibrosis.

## Materials and methods

### Study population

NHANES is a long-term, large-scale health survey program initiated and conducted every 2 years by the National Center for Health Statistics (NCHS), a division of the Centers for Disease Control and Prevention (CDC). Through a complex, multistage sampling design, NHANES collects health and nutrition data representative of the United States population, including personal interviews, physical examinations, and laboratory test results. NHANES data are widely used to study public health trends, assess the burden of disease and nutritional status, and are freely available to researchers worldwide.

This study analyzed pre-epidemic data from the 2017–2020 NHANES survey, which initially included 15,560 participants. From this cohort, 8,317 individuals aged 18 years and older with vibration-controlled transient elastography (VCTE) results were selected. We excluded 219 participants with unreliable VCTE measurements (liver stiffness quartile/median ratio ≥ 30%), 283 with hepatitis B or C, 821 with excessive alcohol intake (defined as more than two standard drinks per day for women and more than three for men), and those with missing data on neutrophil or high-density lipoprotein cholesterol (HDL-C). Consequently, 6,526 participants were included in the final analysis.

### Variables

Demographic and clinical data were extracted from the NHANES database. Age, gender, race, educational level, body mass index (BMI), diabetes, hypertension, history of cardiovascular disease (CVD), smoking status, and laboratory variables were included. Diabetes mellitus was defined as HbA1c ≥ 6.5% or fasting glucose ≥126 mg/dL; in addition, participants had diabetes if they answered “yes” to any of the following questions: “Do you use insulin?” or “Has your doctor told you that you have diabetes?” or “Do you take glucose-lowering medication?,” Hypertension was defined as a mean systolic blood pressure ≥ 140 mmHg or a mean diastolic blood pressure ≥ 90 mmHg on three consecutive measurements, and participants who responded to the questions “Have you been told you have high blood pressure on two or more occasions” or “Do you have to take prescription medication for high blood pressure?” A “yes” response was also defined as hypertension. A history of cardiovascular disease was described as a response confirming a physician’s diagnosis of myocardial infarction, angina pectoris, coronary heart disease, congestive heart failure, or stroke. Smoking status was categorized as a smoker or never smoker based on having smoked fewer than 100 cigarettes in their lifetime.

Laboratory tests included measurements of alanine aminotransferase (ALT), aspartate aminotransferase (AST), total cholesterol (TC), plasma triglycerides (TG), uric acid, albumin (Alb), glycosylated hemoglobin (HbA1c), *γ*-glutamyltranspeptidase (GGT) and high-density lipoprotein cholesterol (HDL-C). NHR was calculated as the ratio of neutrophil counts (10^9^ cells /L) to HDL-C level (mmol/L).

#### The definition of NAFLD, MASLD, and liver fibrosis

NHANES staff use the FibroScan 502 Touch device to assess liver stiffness and fat content. The device measures liver elasticity and stiffness through vibration-controlled transient elastography (VCTE) technology to help determine the extent of liver fibrosis. At the same time, the device measures hepatic steatosis by ultrasound attenuation and records the Controlled Attenuation Parameter (CAP) as an indicator of hepatic fat content. A CAP value of ≥ 274 dB/m was defined as hepatic steatosis according to previous studies, and a CAP value of ≥ 302 dB/m indicated severe hepatic steatosis (17, 18). In addition, liver stiffness measurements (LSM) of ≥ 8.2 kPa, ≥ 9.7 kPa, and ≥ 13.7 kPa represented the F2 (significant fibrosis), F3 (advanced fibrosis), and F4 (cirrhosis) stages of liver fibrosis, respectively (19). NAFLD was defined as the presence of hepatic steatosis in the absence of excessive alcohol consumption or the absence of other chronic liver diseases. MASLD is defined as the presence of hepatic steatosis in the absence of other causes of hepatic steatosis or excessive alcohol consumption and meets at least one of the following adult cardiometabolic criteria (3): (1) BMI ≥ 25 kg/m^2^ or WC > 94 cm for males and > 80 cm for females; (2) fasting serum glucose ≥ 5.6 mmol/L (100 mg/dL) or 2-h post-load glucose level ≥ 7.8 mmol/L (140 mg/dL) or HbA1c ≥ 5.7% (39 mmol/L) or type 2 diabetes or treatment for type 2 diabetes. (3) Blood pressure ≥ 130/85 mmHg or specific antihypertensive drug treatment; (4) TG ≥ 1.70 mmol/L (150 mg/dL) or lipid-lowering treatment; (5) HDL-C ≤ 1.0 mmol/L (40 mg/dL) for males and ≤ 1.3 mmol/L (50 mg/dL) for females or lipid-lowering treatment.

### Statistical analysis

Considering NHANES’s complicated multistage sampling design, sample weights were applied in all analyses to ensure that the results were representative of the US population. Participants were divided into four subgroups according to NHR quartiles. Continuous variables were reported as weighted mean and standard error, while categorical variables were reported as unweighted counts and weighted percentages. One-way analysis of variance (ANOVA) for continuous variables and weighted chi-squared tests for categorical variables were used to compare differences between the four subgroups.

NHR was analyzed as a continuous and categorical variable, with exposure variables grouped by quartiles (the first quartile served as the reference group). Outcome variables included CAP, NAFLD/MASLD, LSM, and liver fibrosis. We used weighted linear regression and weighted logistic regression models for the analysis. In addition, we assessed potential non-linear associations between NHR and the prevalence of NAFLD/MASLD and liver fibrosis using restricted cubic spline (RCS) analysis. The RCS model was adjusted for several confounders, including age, gender, race, smoking status, diabetes, hypertension, CVD, BMI, TC, ALT, and uric acid. Subgroup analyses were conducted by stratifying participants according to age, gender, BMI, hypertension, diabetes, smoking status, and CVD. All data analyses were done using R software (version 4.4.0), and the statistical significance level was set at *p* < 0.05.

## Results

### Baseline characteristics of study participants

A total of 6,526 participants were included in this study. The mean age of the participants was 47.81 ± 17.82 years, with 49.6% males and 50.4% females. The weighted prevalence of NAFLD and MASLD was 44.1 and 43.7%, respectively, 8.9% of participants had liver fibrosis. The baseline characteristics of the study population, grouped according to quartiles of NHR, are presented in Table 1.

**Table 1:** Baseline characteristics of study participants (grouped according to NHR quartile).

Variables	Total (*N* = 6,526)	Q1 (*N* = 1,631, NHR < 2.07)	Q2 (*N* = 1,637, 2.07 ≤ NHR < 3)	Q3 (*N* = 1,627, 3 ≤ NHR < 4.2)	Q4 (*N* = 1,631, NHR ≥ 4.2)	*p* value
Age (year)	47.81 ± 17.82	49.24 ± 17.68	48.48 ± 17.91	47.61 ± 18.02	46.14 ± 17.52	0.008
Gender, *n* (%)						<0.001
Male	3,251 (49.6%)	641 (36.3%)	776 (46.1%)	878 (53.8%)	956 (60.0%)	
Female	3,275 (50.4%)	990 (63.7%)	861 (53.9%)	749 (46.2%)	675 (40.0%)	
Race, *n* (%)						<0.001
Non-Hispanic White	2,210 (61.2%)	422 (56.9%)	537 (61.0%)	590 (63.5%)	661 (62.8%)	
Non-Hispanic Black	1,610 (10.8%)	679 (20.5%)	408 (11.3%)	309 (7.5%)	214 (5.4%)	
Hispanic	711 (7.9%)	134 (6.4%)	160 (7.2%)	220 (9.1%)	197 (8.6%)	
Others	1,995 (20.1%)	396 (16.2%)	532 (20.5%)	508 (19.9)	559 (23.3%)	
Education level, *n* (%)						<0.001
Less than high school	1,215 (11.6%)	211 (7.0%)	312 (12.0%)	331 (12.1%)	361 (14.8%)	
High school or above high school	4,977 (85.2%)	1,345 (90.5%)	1,243 (85.1%)	1,207 (83.7%)	1,182 (82.0%)	
Others	334 (3.2%)	75 (2.5%)	82 (2.9%)	89 (4.1%)	88 (3.2%)	
Hypertension, *n* (%)						<0.001
Yes	2,700 (36%)	597 (28.6%)	674 (34.6%)	711 (37.4%)	718 (42.5%)	
No	3,826 (64%)	1,034 (71.4%)	963 (65.4%)	916 (62.6%)	913 (57.5%)	
Diabetes, *n* (%)						<0.001
Yes	1,321 (15.7%)	178 (6.7%)	295 (11.4%)	371 (17.7%)	477 (25.6%)	
No	5,205 (84.3%)	1,453 (93.3%)	1,342 (88.6%)	1,256 (82.3%)	1,154 (74.4%)	
History of CVD, *n* (%)						0.003
Yes	715 (9.1%)	140 (6.0%)	158 (8.1%)	192 (10.0%)	225 (11.8%)	
No	5,811 (90.9%)	1,491 (94.0%)	1,479 (91.9%)	1,435 (90.0%)	1,406 (88.2%)	
Smoking status, *n* (%)						<0.001
Yes	2,578 (41.4%)	520 (32.1%)	615 (39.0%)	670 (44.5%)	773 (48.8%)	
No	3,948 (58.6%)	1,111 (67.9%)	1,022 (61.0%)	957 (55.5%)	858 (51.2%)	
BMI (kg/m^2^), *n* (%)												<0.001
<25	1,752 (27.2%)	697 (48.2%)	487 (29.6%)	343 (20.3%)	225 (13.6%)	
25−30	2,105 (31.6%)	511 (32.0%)	580 (35.9%)	542 (30.5%)	472 (28.2%)	
≥ 30	2,669 (41.2%)	423 (19.8%)	570 (34.5%)	742 (49.2%)	934 (58.2%)	
ALT (U/L)	22.42±16.91	19.49±16.52	20.87±14.81	23.24±15.92	25.62±19.34	<0.001
AST (U/L)	21.46±11.36	22.23±13.88	21.11±10.24	21.27±10.79	21.31±10.46	0.255
TG (mmol/L)	1.61±1.20	1.07±0.63	1.34±0.69	1.68±1.18	2.26±1.59	<0.001
TC (mmol/L)	4.83±1.05	4.99±1.03	4.81±1.01	4.78±1.01	4.76±1.11	<0.001
Uric acid (mg/dl)	5.35±1.43	4.93±1.37	5.21±1.33	5.48±1.43	5.73±1.45	<0.001
Alb (g/L)	41.09±3.22	41.17±3.08	41.31±3.15	41.18±3.23	40.73±3.39	0.017
HbA1c (%)	5.69 ±0.96	5.48±0.63	5.61±0.84	5.70±0.90	5.95±1.26	<0.001
GGT (U/L)	28.88±37.46	26.23±38.25	27.09±43.05	29.70±36.37	32.07±31.33	0.001
HDL-C (mmol/L)	1.37±0.40	1.77±0.42	1.45±0.31	1.26±0.24	1.06±0.26	<0.001
NHR	3.45±1.85	1.57 ±0.36	2.54±0.27	3.57±0.34	5.85±1.78	<0.001
CAP (dB/m)	264.85±63.13	235.40±53.65	253.76±57.30	274.95±62.05	290.78±64.17	<0.001
LSM (kPa)	5.78±4.69	5.17±2.72	5.26±3.57	5.98±5.06	6.61±6.22	<0.001
NAFLD, *n* (%)	2,839 (44.1%)	427 (24.2%)	615 (37.3%)	804 (50.1%)	993 (61.6%)	<0.001
MASLD, *n* (%)	2,813 (43.7%)	413 (23.3%)	608 (37.1%)	801 (49.9%)	991 (61.3%)	<0.001
Severe hepatic steatosis, *n* (%)	1,850 (29.0%)	224 (12.5%)	359 (21.0%)	549 (34.6%)	718 (45.2%)	<0.001
Liver fibrosis, (%)	610 (8.9%)	82 (4.0%)	126 (6.1%)	177 (10.6%)	225 (13.9%)	<0.001
F2, *n* (%)	233 (3.4%)	33 (1.5%)	55 (2.9%)	74 (4.5%)	71 (4.3%)	0.005
F3, *n* (%)	214 (3.0%)	27 (1.3%)	41 (2.1%)	55 (2.6%)	91 (5.6%)	<0.001
F4, *n* (%)	163 (2.5%)	22 (1.2%)	30 (1.1%)	48 (3.6%)	63 (3.9%)	0.001

Continuous variables are presented as mean ± SE; categorical variables are presented as unweighted counts (weighted percentages). CVD, cardiovascular disease; BMI, Body mass index; ALT, Alanine aminotransferase; AST, Aspartate aminotransferase; TG, Triglyceride, TC, Total cholesterol; Alb, Albumin; GGT, γ-glutamyltranspeptidase; HDL-C, High-density lipoprotein cholesterol; NHR, Neutrophil/High-Density Lipoprotein Cholesterol Ratio; CAP, Controlled attenuation parameter; HbA1c, Glycosylated hemoglobin A1c; LSM, Liver stiffness measurement; NAFLD, Nonalcoholic fatty liver disease; MASLD, metabolic dysfunction-associated steatotic liver disease.

The results showed that the prevalence of NAFLD, MASLD, severe hepatic steatosis, liver fibrosis, and the severity of hepatic fibrosis gradually increased with increasing NHR (*p* < 0.05). In addition, it was found that there were significant differences between participants with different NHR levels in terms of age, gender, race, education level, hypertension, diabetes, smoking status, history of CVD, BMI, TG, TC, ALT, Alb, GGT, HbA1c, HDL-C, and uric acid (*p* < 0.05).

### Association of NHR with NAFLD/MASLD

As shown in Table 2, we analyzed the effect of NHR on CAP and NAFLD/MASLD using weighted multiple regression models adjusted for all possible confounding variables (age, gender, race, smoking, diabetes, hypertension, history of CVD, BMI, TC, ALT, and uric acid).

**Table 2:** Association of NHR with NAFLD/MASLD.

	Model 1: *β*/OR (95% CI) *p* value	Model 2: *β*/OR (95% CI) *p* value	Model 3: *β*/OR (95% CI) *p* value
CAP (dB/m)			
NHR	9.7 (7.6–12) <0.001	9.3 (7.1–11) <0.001	2.5 (0.51–4.5) 0.019
NHR (Quartile)			
Q1	Reference	Reference	Reference
Q2	18 (13–24) <0.001	17 (12–22) <0.001	3.0 (−1.6–7.5) 0.175
Q3	40 (34–46) <0.001	38 (33–43) <0.001	11 (8.1–15) <0.001
Q4	55 (50–61) <0.001	54 (48–59) <0.001	17 (12–22) <0.001
P for trend	15 (14–16) <0.001	14 (13–16) <0.001	4.8 (3.4–6.1) <0.001
NAFLD			
NHR	1.35 (1.28, 1.43) <0.001	1.35 (1.28–1.44) <0.001	1.11 (1.01–1.22) 0.029
NHR (Quartile)			
Q1	Reference	Reference	Reference
Q2	1.87 (1.51–2.32) <0.001	1.84 (1.44–2.35) <0.001	1.24 (0.94–1.63) 0.105
Q3	3.14 (2.58–3.83) <0.001	3.15 (2.59–3.82) <0.001	1.52 (1.24–1.86) 0.002
Q4	5.03 (4.14–6.11) <0.001	5.14 (4.04–6.55) <0.001	2.00 (1.46–2.75) <0.001
P for trend	1.52 (1.45–1.59) <0.001	1.53 (1.45–1.62) <0.001	1.20 (1.11–1.30) <0.001
MASLD			
NHR	1.36 (1.28–1.44) <0.001	1.36 (1.28–1.45) <0.001	1.12 (1.02–1.23) <0.001
NHR (Quartile)			
Q1	Reference	Reference	Reference
Q2	1.87 (1.51–2.32) <0.001	1.92 (1.49–2.48) <0.001	1.29 (0.97–1.72) 0.071
Q3	3.14 (2.58–3.83) <0.001	3.32 (2.76–3.99) <0.001	1.59 (1.32–1.91) <0.001
Q4	5.03 (4.14–6.11) <0.001	5.41 (4.21–6.95) <0.001	2.09 (1.51–2.89) <0.001
P for trend	1.53 (1.46–1.60) <0.001	1.55 (1.46–1.63) <0.001	1.21 (1.12–1.31) <0.001

Model 1: no covariates were adjusted.

Model 2: adjusted for age, gender, race.

Model 3: adjusted for age, gender, race, smoking, diabetes, hypertension, history of CVD, BMI, TC, ALT, and uric acid.

We performed weighted linear regression analyses with CAP as the outcome. When NHR was included as a continuous variable in the model for analysis, the results showed that CAP increased by 2.5 dB/m for each unit increase in NHR in the fully adjusted model (*β* = 2.5; 95% CI (0.51–4.5); *p* = 0.019). NHR was included as a categorical variable (quartiles) in the analysis model, and after adjusting for all confounding variables, CAP values increased significantly with higher levels of NHR (P for trend <0.001), with participants in the fourth quartile group of NHR having the highest CAP values compared with the first quartile of NHR (*β* = 17; 95% CI (12–22); *p* < 0.001).

Subsequently, we performed weighted logistic regression analyses with NAFLD and MASLD as outcomes, which showed that higher NHR was associated with a higher prevalence of NAFLD and MASLD. After fully adjusting for confounding variables, each unit increase in NHR was associated with an 11% (OR = 1.11; 95% CI (1.01–1.22); *p* < 0.05) and 12% (OR = 1.12; 95% CI (1.02–1.23); *p* < 0.001) increase in the prevalence of NAFLD and MASLD, respectively. The inclusion of NHR as a categorical variable (quartiles) in the analysis model showed that the prevalence of NAFLD and MASLD increased with increasing levels of NHR after adjusting for all confounding variables (P for trend <0.001), and participants in the fourth quartile group of NHR had the highest risk of NAFLD and MASLD compared with the first quartile of NHR.

### Relationship between NHR and liver fibrosis

Similarly, we analyzed the effect of NHR on LSM and liver fibrosis using weighted multivariate regression models. As presented in Table 3, the unadjusted model indicated that each unit increase in NHR was associated with a 0.29 kPa increase in LSM (beta = 0.29; 95% CI: 0.22, 0.37; *p* < 0.001) and a 21% higher risk of liver fibrosis (OR = 1.21; 95% CI: 1.24, 1.29; *p* < 0.001). However, after adjustment for all confounders, the association between higher NHR and liver fibrosis was no longer statistically significant (Table 3).

**Table 3:** Relationship between NHR and liver fibrosis.

	Model 1: *β*/OR (95% CI) *p* value	Model 2: *β*/OR (95% CI) *p* value	Model 3: *β*/OR (95% CI) *p* value
LSM (kPa)			
NHR	0.29 (0.22–0.37) <0.001	0.28 (0.19–0.36) <0.001	0.07 (−0.04–0.18) 0.171
NHR (Quartile)			
Q1	Reference	Reference	Reference
Q2	0.09 (−0.26–0.43) 0.604	0.07 (−0.28–0.41) 0.693	−0.31 (−0.64–0.01) 0.058
Q3	0.81 (0.40–1.2) <0.001	0.76 (0.38–1.1) <0.001	−0.02 (−0.38–0.34) 0.896
Q4	1.4 (1.1–1.8) <0.001	1.4 (1.0–1.8) <0.001	0.27 (−0.24–0.78) 0.253
P for trend	0.42 (0.32–0.52) <0.001	0.41 (0.30–0.52) <0.001	0.11 (−0.03–0.25) 0.10
Liver fibrosis			
NHR	1.21 (1.24–1.29) <0.001	1.22 (1.13–1.31) <0.001	1.05 (0.98–1.13) 0.178
NHR (Quartile)			
Q1	Reference	Reference	Reference
Q2	1.58 (1.01–2.47) 0.043	1.58 (1.00–2.51) 0.051	1.07 (0.60–1.90) 0.801
Q3	2.89 (1.92–4.35) <0.001	2.90 (1.91–4.41) <0.001	1.42 (0.85–2.39) 0.157
Q4	3.91 (2.75–5.57) <0.001	4.01 (2.77–5.80) <0.001	1.49 (0.84–2.63) 0.145
P for trend	1.40 (1.31–1.50) <0.001	1.41 (1.32–1.51) <0.001	1.11 (0.98–1.25) 0.086

Model 1: no covariates were adjusted.

Model 2: adjusted for age, gender, race.

Model 3: adjusted for age, gender, race, smoking, diabetes, hypertension, history of CVD, BMI, TC, ALT, and uric acid.

### Potential non-linear relationship between NHR and NAFLD/MASLD and liver fibrosis

**Figure 1:**
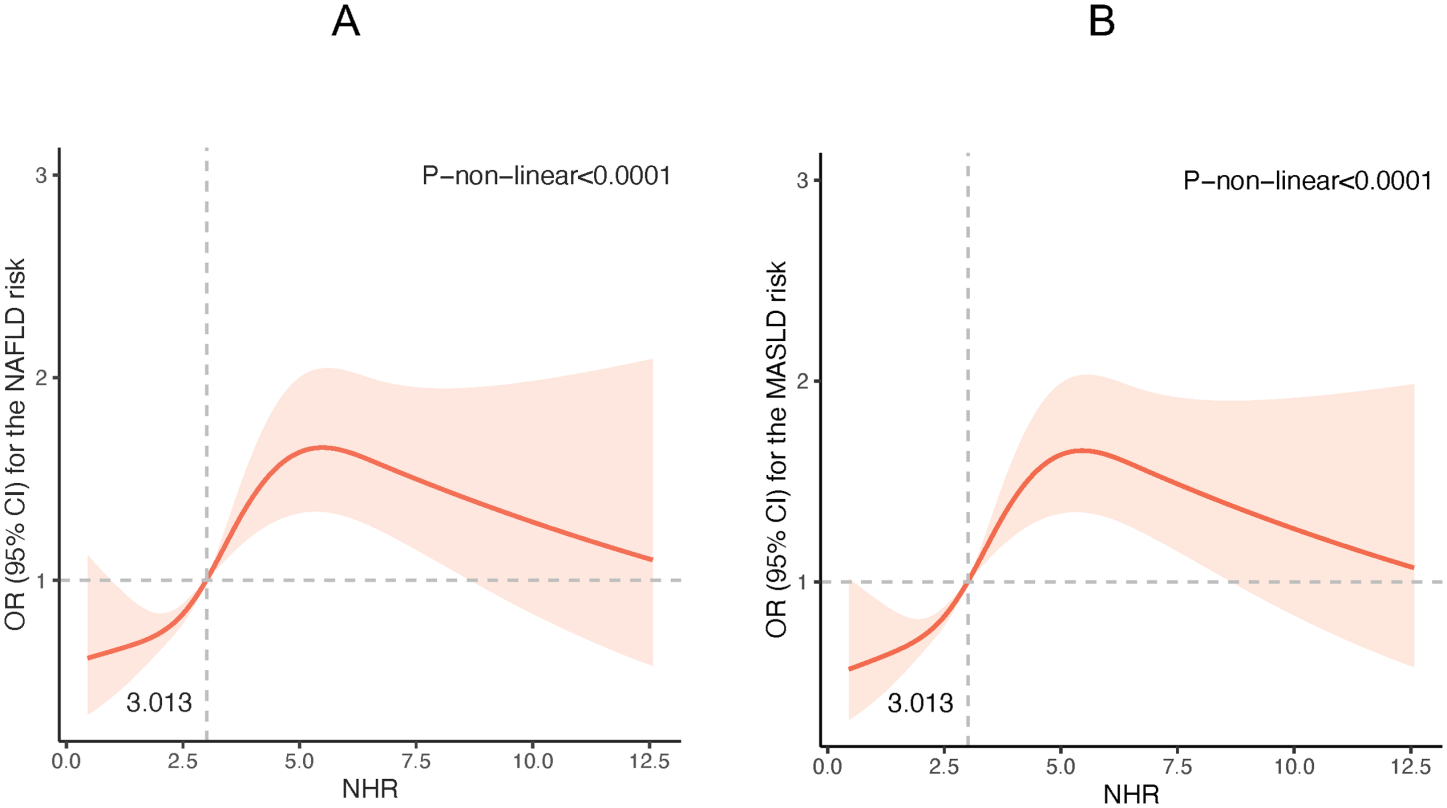
Restricted cubic spline (RCS) plot of the non-linear relationship between NHR and NAFLD /MASLD risk. **(A)** NHR - NAFLD; **(B)** NHR – MASLD. (Nonlinear relationships were detected after age, gender, race, smoking, diabetes, hypertension, history of CVD, BMI, TC, ALT, and uric acid).

**Figure 2:**
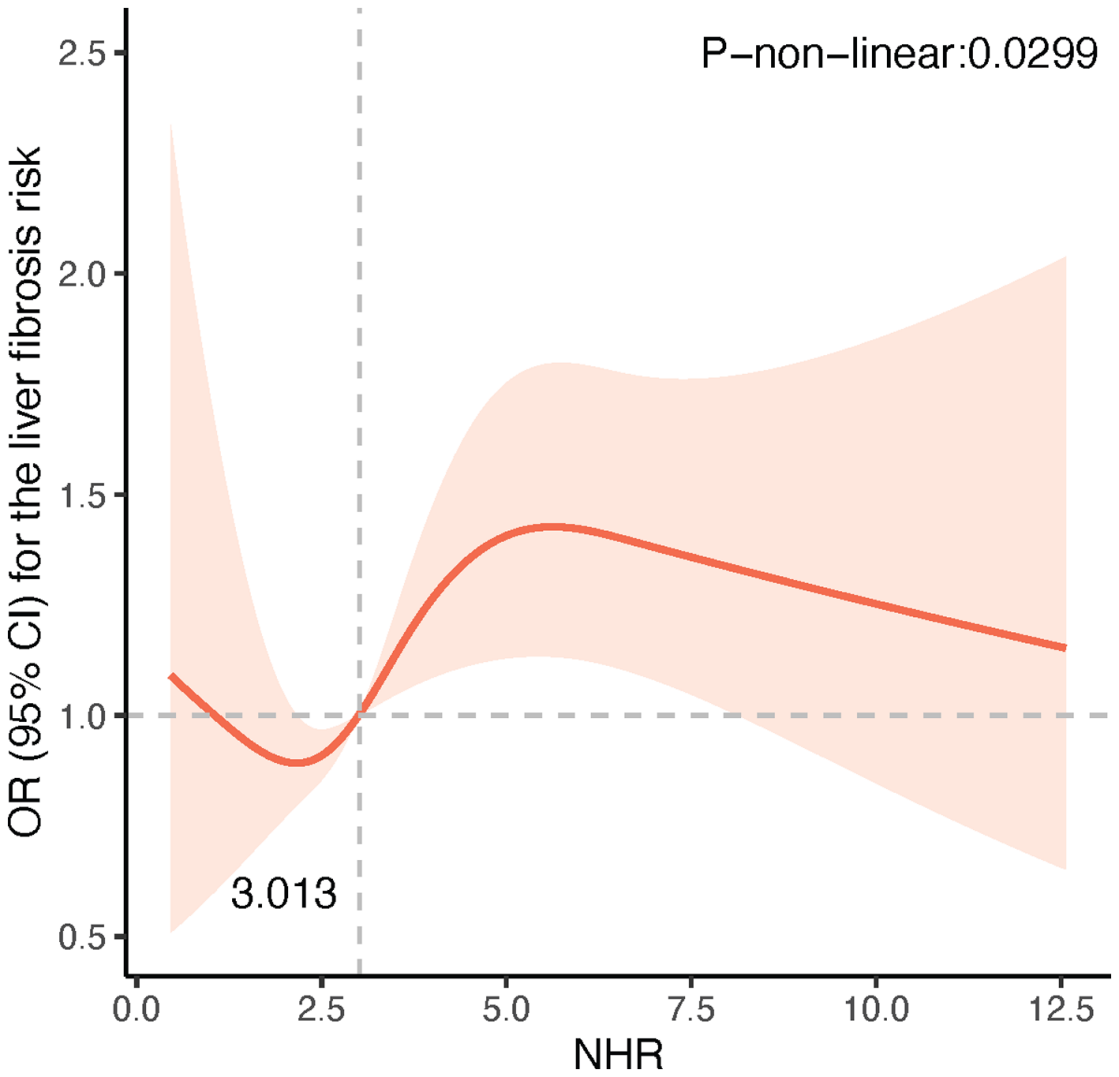
Restricted cubic spline (RCS) plot of the non-linear relationship between NHR and risk of liver fibrosis. A nonlinear relationship was detected after age, gender, race, smoking, diabetes, hypertension, history of CVD, BMI, TC, ALT, and uric acid.

The potential non-linear associations between NHR and NAFLD/MASLD and liver fibrosis were analyzed using the RCS model. As shown in Figure 1, the NHR was nonlinearly linked with the prevalence of NAFLD and MASLD (P-non-linear <0.001). Figure 1 reveals that at NHR < 3.013, the smaller the NHR, the lower the risk of NAFLD and MASLD. Figure 2 shows that lower NHR levels were not significantly associated with the risk of liver fibrosis, but the risk of liver fibrosis increased significantly when the NHR exceeded 3.013.

### Subgroup analysis

**Figure 3:**
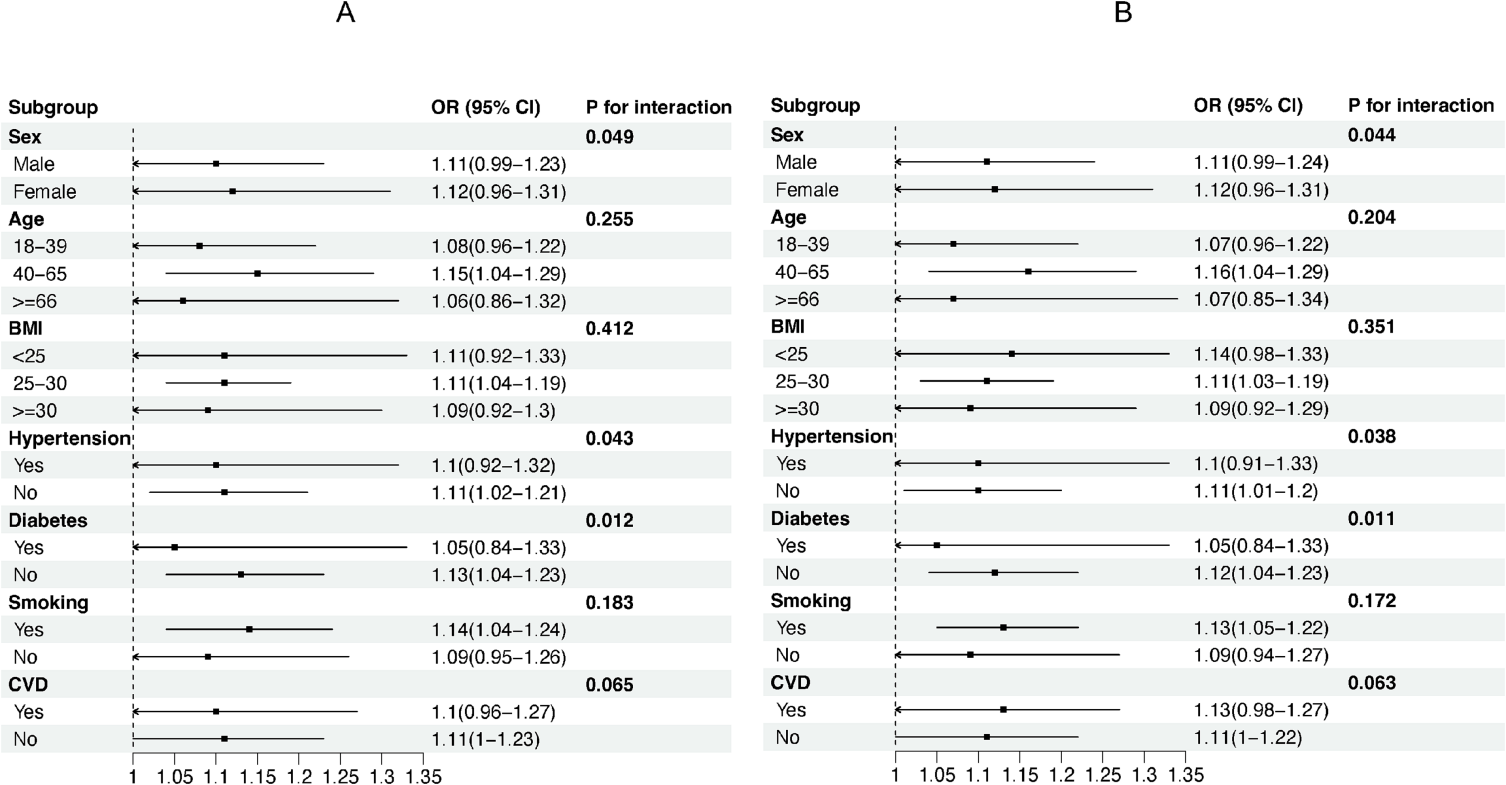
Subgroup analyses of the association between NHR and NAFLD/MASLD. **(A)** NHR - NAFLD; **(B)** NHR - MASLD.

**Figure 4:**
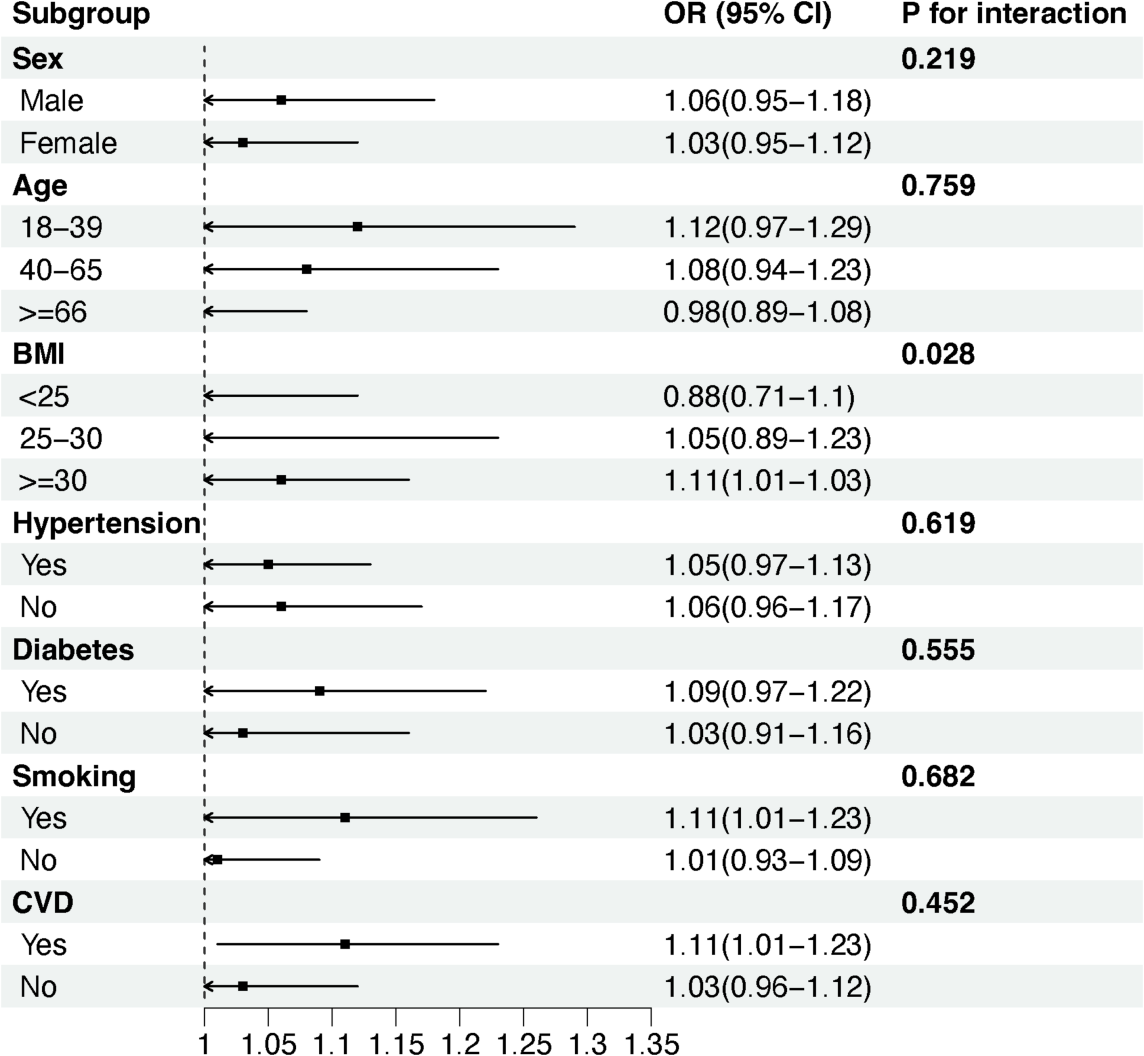
Subgroup analysis of the association between NHR and liver fibrosis.

We used stratified weighted multiple regression analysis to investigate the association of NHR with NAFLD/MASLD and liver fibrosis in different populations, dividing participants into subgroups based on gender, age, BMI, hypertension, diabetes, smoking status, and history of CVD for the analyses and interaction tests as shown in Figure 3, there was a significant interaction between NHR and gender, hypertension, and diabetes (P for interaction <0.05), and the positive correlation between NHR and NAFLD/MASLD was stronger in female, non-diabetic, and hypertensive participants. In addition, BMI was the factor that significantly interacted with the relationship between NHR and hepatic fibrosis in different population subgroups, and the positive association between NHR and hepatic fibrosis was more pronounced, especially in the obese population (BMI ≥ 30) (Figure 4).

## Discussion

This study evaluated the association between NHR and NAFLD, MASLD, and liver fibrosis in the American population. The results of the study showed that NHR was positively and nonlinearly associated with NAFLD and MASLD. In addition, although lower NHR levels were not significantly associated with the risk of liver fibrosis, the risk of liver fibrosis increased significantly when NHR exceeded 3.013. Subgroup analyses further revealed that the association between NHR and NAFLD/MASLD was more significant in women and individuals without hypertension and diabetes, and the association between NHR and liver fibrosis was more prominent in participants with BMI >30.

Our study extends and supports previous findings. An earlier study involving 936 individuals from a Chinese population demonstrated that NHR was positively associated with the risk of ultrasound-diagnosed NAFLD, suggesting that NHR may be a valid predictor of NAFLD (20). Our study validated this association and assessed the prevalence and severity of NAFLD using data from a large-scale U.S. general population, incorporating the VCTE technology of the FibroScan device. Studies have shown that the accuracy of VCTE in diagnosing hepatic steatosis and fibrosis is comparable to liver biopsy (21, 22), which enhances the broad applicability and reliability of the results.

The main features of NAFLD/MASLD include hepatic lipid accumulation, inflammatory response, fibrosis formation, and hepatocyte injury (2). Hepatic steatosis is the pathological basis for the progression of NAFLD/MASLD, often accompanied by the onset of chronic inflammatory responses (23). Studies have shown that chronic inflammation plays a vital role in the development of NAFLD/MASLD (24, 25). Notably, chronic inflammation in the liver may result in dysfunction of hepatic sinusoidal endothelial cells and vascular endothelium, leading to an increased risk of cardiovascular disease, which is not directly related to the presence or absence of diabetes mellitus, hypertension, and obesity (26). An analysis based on NHANES 2017–2018 showed that the neutrophil-to-albumin ratio (NPAR), a systemic marker of inflammation, was significantly associated with NAFLD and advanced liver fibrosis (27). When immune cells such as neutrophils and lymphocytes are activated, they release pro-inflammatory cytokines that promote the development of NAFLD (28). Neutrophils are the first immune cells to respond to inflammation, producing cytokines to promote lymphocyte activation and recruit macrophages, ultimately leading to chronic inflammation (29, 30). In addition, HDL-C reduces neutrophil activation, adhesion, spreading, and migration, inhibiting oxidized LDL production and exerting anti-inflammatory and antioxidant effects (31, 32). Therefore, NHR, as a combination of neutrophil numbers and HDL-C levels, may reflect the state of chronic inflammation and oxidative stress and serve as a sensitive indicator of the pathological process of NAFLD/MASLD.

Recent studies have shown that NHR is associated with the progression of several diseases, particularly cardiovascular and metabolic diseases (13, 14, 33), Our analysis revealed a non-linear association of NHR with NAFLD and liver fibrosis, which may be related to the complex interaction of neutrophils and HDL-C in the development of metabolic diseases. In *in vitro* experiments, mice fed a high-fat diet showed increased neutrophil infiltration in the liver, accompanied by the development of hepatic steatosis and inflammation. Neutrophil depletion in mice using the 1A8 antibody significantly reduced liver triglyceride accumulation, hepatic inflammation, and fibrosis (34). Thus, NHR may play an essential role in the progression of NAFLD/MASLD, with high NHR levels strongly associated with increased severity of hepatic steatosis and fibrosis.

In particular, the risk of liver fibrosis increases significantly when the NHR exceeds 3.013, probably due to exacerbation of chronic inflammation. Neutrophil accumulation has been linked to the progression of liver fibrosis and cirrhosis. During the development of liver inflammation and fibrosis, immature neutrophils with pro-inflammatory properties are released into the circulation, further exacerbating the inflammatory response and liver fibrosis (35). In addition, several studies have shown that neutrophil elastase (NE), neutrophil granule protein (PR3), tissue protease G (CSTG), and other neutrophil-derived proteases play a critical role in the progression of hepatic steatosis and inflammation in NAFLD (36–38). The role of myeloperoxidase (MPO) in the progression of liver fibrosis has also been demonstrated (39). These mechanisms may explain the association between NHR and hepatic steatosis and fibrosis.

Subgroup analyses in this study revealed a significant interaction between gender and the association of NHR with NAFLD/MASLD, The positive correlation between NHR and NAFLD/MASLD was more pronounced in the female population. This gender difference may be attributed to the unique physiological and endocrine characteristics of females. Studies have shown that higher levels of estrogen in women can exert anti-inflammatory and antioxidant effects through multiple pathways and to some extent, mitigate the liver damage caused by inflammatory responses and lipid metabolism disorders (40). However, after menopause, this protective effect is weakened by decreasing levels of estrogen, and the inflammatory response is relatively enhanced (41), which may make the relationship between NHR and NAFLD/MASLD more significant. Additionally, the association between the NHR and NAFLD/MASLD was modified by hypertension and diabetic status, both of which are recognized risk factors for the development and progression of NAFLD/MASLD (42, 43), They also strongly correlate with systemic inflammation and disorders of lipid metabolism (44, 45). Therefore, co-morbid diabetes and hypertension may mask the association between NHR and NAFLD/MASLD. In the obese population, the NHR index was significantly associated with the risk of liver fibrosis, which may be due to the accumulation of fat and metabolic abnormalities caused by obesity, which triggered a series of inflammatory reactions and then promoted the development of liver fibrosis (46), making NHR an important influence factor of liver fibrosis in the state of obesity.

### Study strengths and limitations

The main strength of this study is the use of large-scale data from the general population of the United States, with a large and nationally representative sample size. In addition, we used VCTE to assess hepatic steatosis and hepatic fibrosis, which provided greater diagnostic accuracy. However, this study has some limitations. Firstly, as a cross-sectional study, it was impossible to establish a causal relationship between NHR and hepatic steatosis and fibrosis. Secondly, although we adjusted for confounders as much as possible, there may still be potential confounding variables that were not considered. Thirdly, although VCTE demonstrated high accuracy in non-invasive diagnosis, its diagnostic accuracy compared to liver biopsy requires further validation. Finally, this study only used data from the US population, and its applicability to Asian populations cannot be directly inferred. Future studies need to include data from different ethnic populations to further validate and expand the findings. In addition, a longitudinal design should be used to assess the long-term association between NHR and NAFLD/MASLD progression and to validate its predictive value.

## Conclusion

In conclusion, this study demonstrated a significant non-linear association between NHR and NAFLD/MASLD and liver fibrosis. NHR can be used as a potential marker for NAFLD/MASLD and liver fibrosis, thus aiding in the early detection and intervention of NAFLD/MASLD.

## Data Availability

Publicly available datasets were analyzed in this study. This data can be found at: https://wwwn.cdc.gov/nchs/nhanes/Default.aspx.
